# How Bees Deter Elephants: Beehive Trials with Forest Elephants (*Loxodonta africana cyclotis*) in Gabon

**DOI:** 10.1371/journal.pone.0155690

**Published:** 2016-05-19

**Authors:** Steeve Ngama, Lisa Korte, Jérôme Bindelle, Cédric Vermeulen, John R. Poulsen

**Affiliations:** 1 Axe Gestion des Ressources Forestières, Département Biosystem Engineering/Ingénierie Des Biosystèmes, Gembloux Agro-Bio Tech, Université de Liège, Gembloux, Belgique; 2 Laboratoire de santé et production animale, Département de Zootechnie, Institut de Recherches Agronomiques et Forestières, Centre National de la Recherche Scientifique et Technologique, Libreville, Gabon; 3 Gabon Biodiversity Program, Center for Conservation Education and Sustainability, Smithsonian Conservation Biology Institute, Gamba, Gabon; 4 Precision Livestock and Nutrition Unit, Gembloux Agro-Bio Tech, University of Liège, Gembloux, Belgique; 5 Nicholas School of the Environment Duke University, Durham, North Carolina, United States of America; CNRS, FRANCE

## Abstract

In Gabon, like elsewhere in Africa, crops are often sources of conflict between humans and wildlife. Wildlife damage to crops can drastically reduce income, amplifying poverty and creating a negative perception of wild animal conservation among rural people. In this context, crop-raiding animals like elephants quickly become “problem animals”. To deter elephants from raiding crops beehives have been successfully employed in East Africa; however, this method has not yet been tested in Central Africa. We experimentally examined whether the presence of *Apis mellifera adansonii*, the African honey bee species present in Central Africa, deters forest elephants (*Loxodonta Africana cyclotis*) from feeding on fruit trees. We show for the first time that the effectiveness of beehives as deterrents of elephants is related to bee activity. Empty hives and those housing colonies of low bee activity do not deter elephants all the time; but beehives with high bee activity do. Although elephant disturbance of hives does not impede honey production, there is a tradeoff between deterrence and the quantity of honey produced. To best achieve the dual goals of deterring elephants and producing honey colonies must maintain an optimum activity level of 40 to 60 bee movements per minute. Thus, beehives colonized by *Apis mellifera adansonii* bees can be effective elephant deterrents, but people must actively manage hives to maintain bee colonies at the optimum activity level.

## Introduction

With human population growth and related livelihood activities, wildlife shares space and resources with people more than ever, resulting in increased interactions and conflicts between them [1,2]. Conflicts, which are among the most visible traits of “human-wildlife” interactions, can lead to injury and even death for both people and wildlife, and are primarily associated with crop raiding in Africa [3]. Crops often attract wildlife because they are more palatable with few secondary defenses compared to wild food sources [4,5,6]. In addition to crops, some wild animals such as elephants have a preference for the secondary vegetation that grows on the borders of rural agricultural landscapes [7,8].

Agriculture is the main amplifier of human-elephants conflicts [9]. As agriculture and human extractive activities grow, habitat loss, resource pressure and human-elephant conflict are expected to increase, contributing to decreases in elephant populations [9,10,11,12]. Forest elephant populations (*Loxodonta africana cyclotis*) in Central Africa declined by 62% between 2002 and 2011 to less than 10% of their potential size [13]. To save the remaining forest elephants, poaching for ivory must be stopped and human elephant conflict must be addressed with efficient and non-lethal deterrents [13,14]. The remaining forest elephants occupy less than 25% of their potential home range [15]. Gabon harbors the majority of the remaining population, even though the country accounts for only 22% of the potential home range of the species [13,15,16]. Thus, rural Gabon villages and their surroundings are potential “hot spots” of human elephant conflicts and crucial in addressing this issue [8,17].

In Gabon, human-elephant conflict is widespread across the country [3,17,18,19]. Elephants easily enter farms, even those close to villages, although they avoid high-traffic roads and areas of intense poaching [3,4,18]. While sporadic, elephant damage is usually severe, sometime resulting in complete destruction of fields [3]. Crop damage has harmful consequences for the livelihoods of local people with losses of crops ranging between 30% and 100% [17,19]. Crop damage also contributes to a negative perception of wildlife, deteriorating support for conservation from local people as they feel powerless to stop the loss of their labor and food [3,17,20].

Local people use a variety of methods to protect crops from elephants in Gabon [3,17,18]. These methods range from erecting scarecrows in fields to building barriers and deterrents such as fires, clearing the perimeters of fields, and setting up metal string fences with noisemakers [3,18,21,22]. Communities also work collectively, grouping fields in a single area that is easier to monitor and protect [18,21,22]. Unfortunately, elephants almost always find ways to overcome these measures [21,22], underscoring the urgent need to develop effective, non-lethal methods, such as beehives, to keep elephants out of fields [23].

The use of beehives to protect plantations has successfully reduced elephant damage on crops in east Africa, making it a promising non-lethal method for reducing human-wildlife conflict [14,24,25]. Beekeeping has the additional advantage of producing honey, potentially diversifying and increasing the livelihoods of local farmers [26]. While promising, this method needs further research because no comparable work has been conducted on forest elephants or with *Apis mellifera adansonii*, the only species of African honey bee in central Africa. In this study, we conduct the first test of whether beehives can deter forest elephants in Central Africa. Specifically, we examine whether forest elephants avoid both empty and active beehives, and whether bee activity influences the rate of elephant attack. Finally, we assess if bees face a tradeoff between defending the hive (deterring elephants) and producing honey.

## Materials and Methods

We conducted this study near the town of Gamba (1°55′S, 9°50′E) in the Gamba Complex of Protected Areas in southwest Gabon. The Gamba Complex consists of two national parks (Loango, 1550 km^2^ and Moukalaba-Doudou, 4500 km^2^) that are longitudinally divided by an ‘industrial corridor’ for oil production called the Rabi-Ndogo Protected Area (3500 km^2^) [27]. Since the start of oil production in 1960, Gamba has grown from a small fishing village to a town of approximately 9,000 people [27]. As in many places in Gabon, human-elephant conflict occurs in Gamba because of the high density of forest elephants in this biodiverse area [3,15,27]. The region experiences bimodal rainfall with a short dry season in January and a long dry season extending from June to August, and rains during the rest of the year [27]. Mean annual rainfall averages 2093 mm, relative humidity is around 85% and average temperature is relatively constant ranging from 24 to 28°C [27].

To investigate whether beehives serve as forest elephant deterrents, we recorded elephant behavior near trees on which we hung beehives and on control trees without beehives. We selected 6 trees of *Irvingia gabonensis* and 4 trees of *Sacoglottis gabonensis*, species whose fruits are commonly consumed by forest elephants [28,29], for a total of ten sites. We received permissions to conduct this research from: (i) the Gabonese Agricultural and Forestry Research Institute, National Center for Scientific Research and Technology (Institut de Recherches Agronomiques et Forestières—Centre National de la Recherche Scientifique et Technologique, IRAF-CENAREST); (ii) the Smithsonian Institution; and, (iii) the Health Safety, and Environment (HSE) Department of Shell Gabon. As our trial did not involve taking samples from forest elephants, a protected species in Gabon, no others specific permits were required. The single species of stinging honey bee species in Gabon is not protected and is traditionally used to produce honey [30]; thus no additional authorizations were necessary to work with the bees, although we obtained authorization from the Smithsonian Institution and the Health Safety, and Environment (HSE) Department of Shell Gabon to use only trees located where human safety was guaranteed.

The trees had similar heights and canopy areas, with signs of recent elephant activity (fresh tracks and dungs) in their vicinities. The trees canopies were large enough (≥10m) so that animals could feed on one side of a tree without disturbing a beehive on the other side. We randomly designated two of the *Irvingia gabonensis* trees and one *Sacoglottis gabonensis* as controls. We hung two empty beehives at each of the 7 remaining experimental trees at a height of approximately 1–1.5 m from the ground. Each hive was treated as an experimental unit, for a total of 14 experimental units. All beehives were constructed from Bilinga wood (*Nauclea dederrichii*) using the same modified Langstroth hive model [31].

To record forest elephant activity, at each site we placed a camera trap (RC55 Rapidfire, Reconyx, Holmen, Wisconsin) at a height of about 1.5m above ground in surrounding trees located 10 to 20m from the focal trees [32,33], setting the time delay for photo capture to two seconds (2s). We measured the distances between fixed objects and our focal trees and beehives so that we could estimate the position of elephants relative to the trees and beehives in the photos. We monitored hives weekly from November 2011 to February 2013 for a total of 70 weeks.

There is a strong positive relationship between beehive activity level and the colony’s defensive ability [34]. Active bees, called “foragers”, are in charge of a colony’s external duties, such as collecting nectar and pollen or guarding the nest [35,36]. To test if bee activity affects beehive efficiency in deterring forest elephants, we recorded video of bees entering and exiting hives between 10am and 2pm on days when it was not raining [34,35,37,38]. We estimated the rate of bee activity as the number of bee movements per minute” (b.mvt/min) using the slowdown speed video mode of a Canon PowerShot S3IS camera. We defined a movement as one bee entering or exiting the hive.

To assess whether there is a tradeoff between defending the hive (i.e. deterring elephants) and producing honey, we harvested honey as often as possible during routine checks of beehives. We always left at least one comb half-filled with honey (≈1 kg) to ensure that bees had sufficient food. Pests can damage honey and bee colonies [39,40], so we removed ants and others parasites from hives whenever they were present. To limit pest access to hives, we cleared vegetation within 2m of the hive and greased the wires supporting them. Despite these efforts, bees often deserted beehives, sometimes after heavy parasitism and predator attacks resulting in the loss of honey (which was not accounted for in the total honey production). Over the course of the experiment, three camera traps malfunctioned in two control trees (*Sacoglottis* and *Irvingia*) and one experimental *Sacoglottis*.

We measured five response variables related to elephant behavior and beehives: (i) elephant visits; (ii) time spent by elephants at sites, estimated as the total number of photo captures multiplied by 2 seconds; (iii) challenges on hives, defined as the number of times an elephant approached within 5m of a hives (counting one challenge per elephant); (iv) disturbed hives, measured as the number of times elephants displaced hives; and (v) the quantity of honey (honey) harvested. We evaluated the effects of several explanatory variables on the response variables, including: (i) presence or absence of fruit, which is related to local seasons; (ii) presence and absence of beehives; (iii) presence and absence of bees; and (iv) bee activity. Bee activity was expressed both quantitatively and qualitatively. Qualitative bee activity levels were expressed as “inactive” (no bees in hives), “low” (<70 b.mvt/min) or “high” (≥70 b.mvt/min) using the natural break point of 70 b.mvt/min observed during our trials. Below, “inactive beehives” refers to beehives with no bees and “active beehives” refers to colonized hives occupied by bees. Active beehives are further characterized as having low bee activity or high bee activity.

For statistical analysis, we first summarized all the data and calculated descriptive statistics. We then focused on factors that would best explain elephant behaviours. Because our data are count data (e.g. numbers of elephant visits), they do not meet the assumptions of parametric, normally distributed data, and are more appropriately modeled by a Poisson distribution or negative binomial distribution (in the case of overdispersion) [41,42]. Therefore, we used generalized linear models (GLM) with these distributions to model the nonlinear relationship between response (number of elephant visits and time spent at sites) and explanatory variables, and to handle overdispersed data with excess zeros [42]. For these models, we treated each week of camera trap observations as a replicate because the presence of bees and fruiting state of trees change over time. Ideally, we would have treated each tree as a random effect to account for site-based characteristics (e.g. maybe a particular tree is easier for elephants to access than other trees) and for possible temporal autocorrelation in the observations. Unfortunately, the low number of trees was not conducive to adding random effects [43]. Akaike’s Information Criterion (AIC) was used to compete models against one another to determine the most parsimonious model that best explained elephant behavior. We performed GLM’s with the lme4 R-package.

To ensure that treating each week of observations as a replicate did not inflate our statistical power and lead to erroneous conclusions, we also analyzed our data as three-way contingency tables with trees as the replicates. We used the Cochran-Mantel-Haenszel statistic to test the hypotheses that elephant visits and time spent at sites varied with fruit presence on the trees, bee presence in hives, and bee activity level. To do so, we classified continuous data into categories, for elephant visits (no visits to a tree, 1 visit, >1 visit), time spent at sites (no time, less than 2 minutes, more than 2 minutes), and bee activity level (no activity, 1–70 b.mvt/min, and >70 b.mvt/min).

In situations where the sample size was too small for GLM’s, we also used Kruskal-Wallis rank sum and Spearman correlation to assess the relationship between explanatory and response variables (i.e elephant challenges on hives, elephants disturbances on hives and honey harvested). We used R version 3.0.3 (R Development Core Team 2014) for all analyses.

## Results

**Fig 1:**
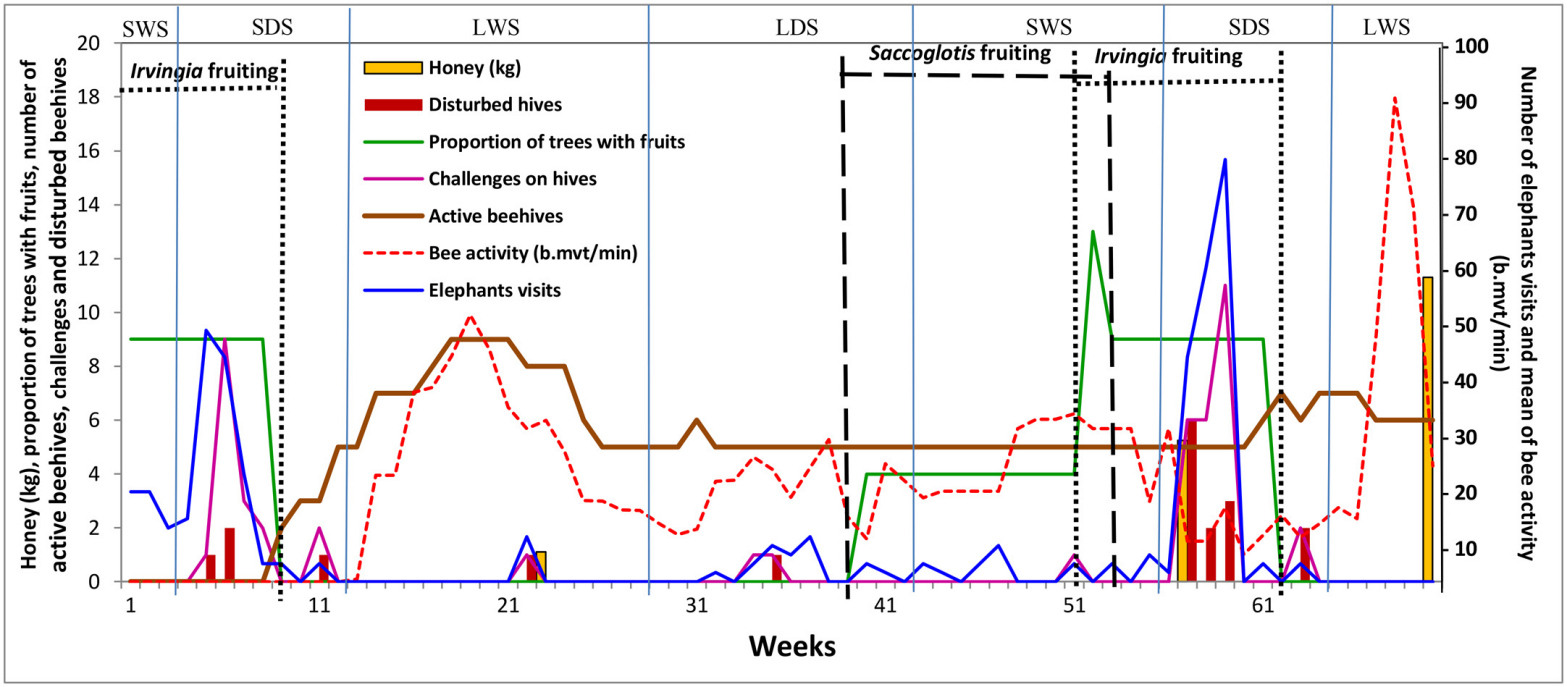
Summary of elephant behaviors and honey collected. SDS, short dry season; LDS, long dry season; SWS, short wet season; LWS, long wet season. Elephant behaviors and measured factors including number of elephants visits, number of challenges on beehives, number of disturbances on hives, number of active beehives, mean of bee activity expressed in bee movement per minute (b.mvt/min) varied according seasons.

During the experiment, we captured 8151 photos of elephants, representing 4h31min42s of time spent by elephants at experimental sites, primarily at night. This includes 255 elephant visits, 46 challenges on beehives (40 on empty hives, 6 on active hives) and 19 beehive disturbances (15 on empty hives, 4 on active hives) (Fig 1). The majority of elephant visits, time spent by elephants at sites, and challenges on beehives were recorded during fruiting periods during the short wet and short dry seasons (Fig 1). The presence of fruits significantly increased the number of elephant visits and time spent by elephants at experimental sites (Table 1). Treating trees as replicates, the frequency of elephant visits was significantly higher with fruit present in trees (17.7% of 0 visits, 54.5% of 1 visits, 89.8% of >1 visits; M^2^ = 139.1, df = 2, p < 0.001). Similarly, the frequency of time elephants spent at the sites was significantly higher when fruit was present in trees (18.9% of 0 time, 57.4% of visits of 2 minutes or less, 89.7% of visits of >2 minutes; M^2^ = 107.5, df = 2, p < 0.001).

**Table 1:** Results of generalized linear models that examined the factors that explain the elephant behaviors.

Independent variables		Response variable: Elephants visits on trees (n = 255)				Response variable: Time spent by elephants in sites (n = 8151photos = 4h 31min 42s)			
Variables	Categories	*estimate*	*SE*	*Z-value*	*p-value*	*estimate*	*SE*	*Z-value*	*p-value*
Presence and absence of fruits (N = 10)	Fruit present	2.9217	0.236	12.37	<0.001	3.04	0.608	5.001	<0.001
Presence and absence of beehives (active and inactive) (N = 10)	Beehives present	-1.2621	0.442	-2.85	0.004	-1.56	0.017	-90.39	<0.001
Presence and absence of Bees (N = 14)	Beehives absent	1.5977	0.473	3.374	<0.001	2.0247	0.023	85.87	<0.001
	Bees absent (inactive beehives)	0.508	0.281	1.804	<0.05	0.674	0.021	31.57	<0.001
Bee activity (N = 14)	Beehives absent	1.417	0.475	2.98	<0.05	1.826	0.023	77.04	<0.001
	Inactive Beehives	0.328	0.311	1.053	0.292	0.476	0.021	22.1	<0.001
	Beehives with High bee activity (active beehives)	-1.875	0.85	-2.21	<0.05	-2.55	0.13	-18.61	<0.001

SE, standard error; inactive beehives are empty or without bees inside; active beehives are colonized by bees.

We set up beehives during the first fruiting period, and they remained empty for 8 weeks (Fig 1). Most elephant visits occurred during *Irvingia* fruiting periods, particularly during the last fruiting period (Fig 1). We recorded the greatest number of active beehives during the long wet season and after fruiting periods (Fig 1). The presence of beehives (active and inactive) significantly reduced elephant activity on sites, with 73% lower visits and 77% less time spent at sites (Table 1)

After 12 weeks, there were a minimum of five active beehives through the end of the trial (Fig 1). For most beehives, bees colonized and deserted the hives multiple times. Compared to active hives, the absence of bees in hives increased elephant visits by 1.6 times, and the absence of beehives increased elephant visits by 5 times (Table 1). The absence of bees also increased time spent by elephants in sites by 2 times, albeit not significantly, and the absence of beehives significantly increased time spent by 7.5 times (Table 1). Treating trees as replicates, the frequency of elephant visits was significantly higher when bees were absent from hives (0 visits– 61.3%, 1 visit– 80.3%, >1 visit 76.9%; M^2^ = 9.2, df = 2, p = 0.010). By contrast, the time spent at trees did not vary significantly (62.4% of 0 mns, 68.5% of visits < 2mns, 82.8% of visits > 2mns; M^2^ = 3.35, df = 2, p = 0.188), although there was a trend towards more time spent when bees were absent from hives.

**Fig 2:**
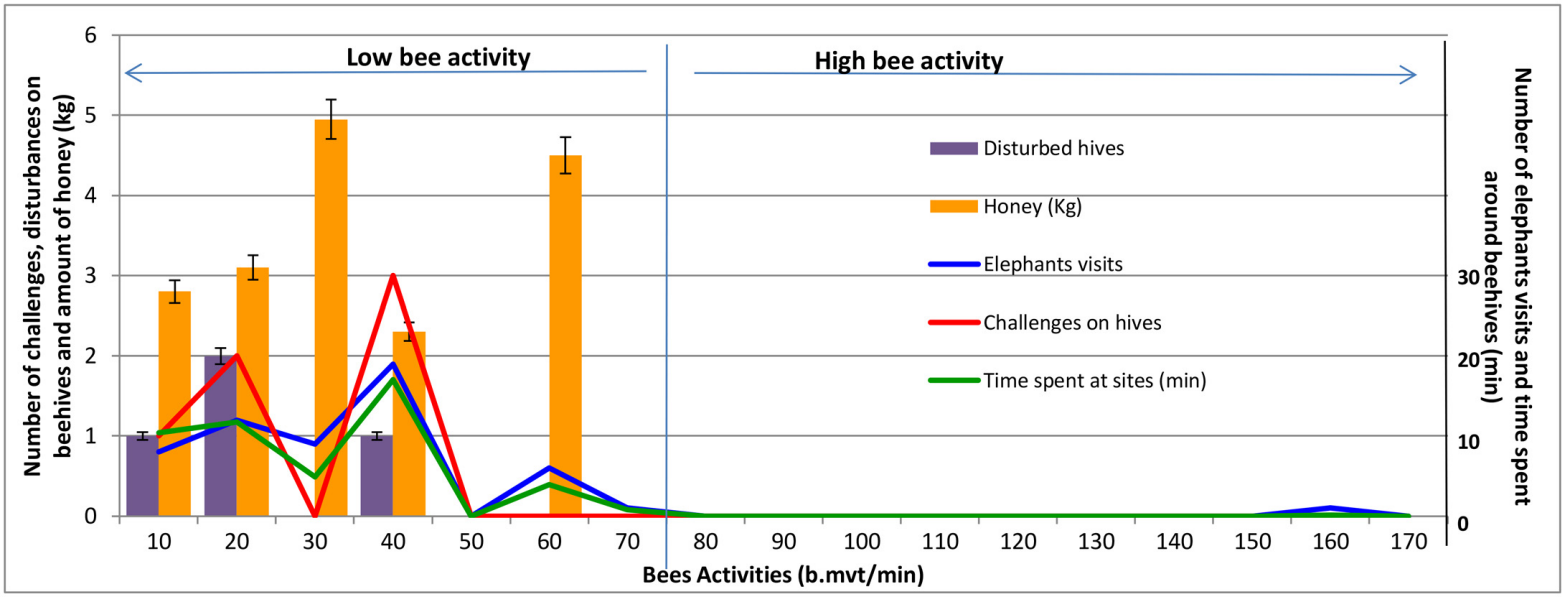
Elephants behavior and honey collected according to bee activity. Bee activity is measured by the number of bees entering and leaving a beehive during one minute (b.mvt/min = number of bee movements per minute). Honey harvested (Honey) was measured in Kg.

Bee activity varied over time and was highest during the long wet season and after fruiting periods (Fig 1). We identified a natural break point in bee activity at 70 b.mvt/min, after what there were no elephant visits, challenges, or disturbances (Fig 2). Compared to beehives with low bee activity, beehives with high bee activity significantly decreased elephant visits by 84% and time spent by elephants by 92% (Table 1). By contrast, at inactive beehives elephant visits were 1.3 times greater and time spent was 1.6 times greater, although the effect on time spent was not statistically significant (Table 1). Treating trees as replicates, the frequency of elephant encounters was significantly higher with lower activity levels (M^2^ = 12.14, d4 = 2, p = 0.016), but the frequency of time spent did not differ with activity level (M^2^ = 6.34, df = 4, p = 0.175).

In addition, the mean number of challenges on sites with active beehives (1.4±0.2) were significantly less than on sites with inactive beehives (5.2±0.5) (Kruskal-Wallis *Χ*^*2*^ = *5*.*94*, *df = 2*, *p = 0*.*05*). Elephants also disturbed more inactive beehives (1.95±0.31) than active ones (0.93±0.00001), although this trend was not significant (Kruskal-Wallis *Χ*^*2*^ = *1*.*03*, *df = 2*, *p = 0*.*596*). Elephants tended to challenge and disturb hives up to a bee activity level of 40 b.mvt/min, but spent less time and visited less often sites with higher bee activity (Fig 2). At bee activity levels of 70 b.mvt/min and greater, elephants almost never visited sites nor interacted with beehives (Fig 2).

When evaluating the tradeoff between protecting sites and producing honey, we found that elephant visits, challenges and disturbances on hives did not stop honey production in beehives (Fig 2); however honey production dropped off at activity levels greater than 70 b.mvt/min. On a monthly basis, 36% of beehives were colonized and accounted for 17.7 kg of honey harvested. Four of these beehives were subject to small beetle (*Aethina tumida*) and ant attacks, resulting in the loss of bee larvae, honey and wax. Only three hives were continuously occupied by bees through the end of the trial. By the 19th week, honey was present in at least one active beehive, but the quantity of honey was most of the time too low to be harvested. At 23 weeks (15 weeks after bees started occupying hives), there was sufficient honey to harvest in some hives (Fig 2). Honey production ranged from 1100 g to 4500 g, representing a mean of 4.1±2.1 kg, per productive beehive. We harvested the greatest quantities (i.e., ≥3kg) from colonies where bee activity ranged from 30 to 60 b.mvt/min and never collected honey in beehives with bee activity ≥70 b.mvt/min (Fig 2). When considering only data from honey harvests, there was a significant positive correlation between bee activity and honey harvested (*r = 0*.*85*, *df = 12*, *p = 0*.*006*).

## Discussion

In the first test of the ability of beehives to deter forest elephants, we found forest elephants in Gabon avoided both empty and active beehives. While the presence of fruits attracted elephants to experimental trees, the presence of bees deterred elephants. We demonstrate that the level of bee activity (low versus high) influences the ability of hives to deter elephants and identify a tradeoff point between beehive defense and honey production.

In Gabon, elephants visited trees with beehives less frequently and spent less time at them compared to trees without beehives, similar to studies in east Africa, where savanna elephants avoid places protected with hives [14,26]. We found that forest elephants can distinguish between empty and occupied beehives, visiting trees with empty hives more often, spending more time feeding at them, and disturbing empty beehives more frequently than active hives. Similarly, Karidozo and Osborn [44] observed bull elephant in Zimbabwe to fell a tree with an empty hive, whereas trees with fully occupied hives were always avoided. Our results suggest that forest elephants, like their savanna congeners, are aware of beehives and able to distinguish between inactive and active beehives. We suspect that the difference between disturbances of empty beehives (15 events) and those with low bee activity (4 events) was not significant because of the small amount of recorded disturbances events (from 255 total visits and 70 trial weeks).

Although forest elephants avoided beehives, they approached and disturbed colonized beehives at fruiting trees four times. This suggests that the food reward of succulent fruit was worth the risk of bee attack. Elephants disturbed beehives with low bee activity and left the sites immediately after disturbing the hives. These observations contradict previous findings that just the hum and smell of occupied hives are sufficient to deter savanna elephants [23,25,45,46], offering some support for studies that question the effectiveness of beehives as elephant deterrents [44,47]. More importantly, our results suggest that elephants may be able to perceive the defense ability of active beehives, avoiding beehives with a high level of activity and challenging those with low bee activity.

We recorded most elephant visits and disturbances at night, which could be a strategy to feed when bees are less active and less able to see and detect elephants [35,37]. Alternatively, it has also been argued that elephants raid crops at night to avoid people [7,26]. Thus, feeding at night could serve to avoid threats such as bees and humans. Beehives with high bee activity were never challenged nor disturbed at night, and bee activity was the most important factor determining the number of elephant visits. Pearce *et al*. [36] and Hunt [38] argue that larger colonies with more foragers are better able to detect threats and defend the nest.

Elephant visits, challenges, and disturbances did not impact honey production. Despite heavy losses due to predators and parasitism, the 4.1kg of honey produced by active beehives in this trial is similar to harvest levels of 4.6kg/hive in Kenya [14]. As would be expected [35,37], we observed a positive correlation between bee activity and honey production until it dropped off at very high bee activity levels: honey production peaked at bee activity levels 30 and 60 b.mvt/min and dropped off at 70 b.mvt/min. According to Smith et *al*. [48], honeybee colonies build drone combs and worker combs for honey storage as well as brood rearing, but these two uses of a cell are mutually exclusive. Therefore, a colony faces a tradeoff between survival (honey storage) and reproduction (brood rearing) in using its combs [48]. Our results suggest that *Apis mellifera adansonii* bees invested first in survival during the low bee activity phases when it was possible to harvest honey. During the high bee activity phase, they shift to reproduction, resulting in low honey storage, but more populated colonies with better defensive ability. Therefore, 70 b.mvt/min might be the point at which survival and reproduction tradeoff for this species. Elephant visits and time spent at sites decreased above 40 b.mvt/min, suggesting that the optimum bee activity for both beehive defense and honey production might be between 40 and 60 b.mvt/min.

Given that the forest elephant population is now less than 10% of its potential size [13], reducing conflict between human and elephants through the use of efficient, non-lethal strategies must be a conservation priority [49]. Our results support previous findings showing bees as a potential solution to both reduce human elephant conflict and enhance the livelihood of local people and wildlife conservation [14,24,26, 50]. Investigations on the economic feasibility of using beehives in plantations are now needed. The strategy of using beehives to deter elephants has several potential benefits for people and their crops. Honey bees are among the best plant pollinators [51, 52], thus the use of beehives in plantations has the potential to increase crop yields as well as the pollination of the surrounding wild plants [52,53]. Erecting beehives in fields also helps maintain the bee population, as traditional practices of beekeeping involve killing the bee colony during honey harvest [53].

As our results suggest, even though elephants could adjust their feeding strategies to overcome the bee threat by feeding at night, bees can recruit more fighters and grow their colonies [38]. Nevertheless, the ability of bees to defend hives from elephants depends on multiple environmental factors. Thus farmers need to manage their beehives to reach the optimum level of elephant defense or honey production, including protecting beehives against predators, parasites and diseases.

We highlight the importance of safety issues when using beehives as a biological non-lethal deterrent. African bees are known for their aggressiveness and the risk they pose to humans [34,54,55]. During the trial, we experienced strong bee aggressions when opening beehives with high bee activity. Bees from these colonies sometimes continued to attack 30 min after honey collection at distances of about 200 m from the experimental sites, with some bees following us even farther and into our vehicle. Karidozo and Osborn [44] in their trial reported that two goats were stung to death after knocking down an occupied hive and that people could not work in nearby fields until the bees calmed down. A single sting to an allergic person and more than 100 stings to non-allergic people can lead to toxic syndrome complications and death [36, 54]. Woyke [34] reported stinging rates of over 200 stings per minute in some colonies of *Apis mellifera adansonii*. As people are encouraged to practice beekeeping as a defense against elephant crop raiding, we underscore the importance of taking the appropriate security measures (i.e., wearing bee suits, gloves and closed shoes, using smokers, etc.) when opening and manipulating a colonized beehive. Bees are less aggressive and more manageable during low bee activity [34]. Although not recommended, working around hives without protective gear should only be done by people without allergies to bee stings and when bee activity is lower than 10 b.mvt/min. We advise people to open beehives with high bee activity only at night, when bees are less active [34]. If beehives with high bee activity are opened during the day, the surrounding areas (about 250m-500m) should be free of people and domestic animals.

The human assault on elephants [13] and the elephant assault on crops [17] are increasing. Mitigating crop raiding by elephants is an important step in changing these trends in Central Africa as human and elephants increasingly share the same spaces. Using beehives will benefit both humans and elephant conservation if properly managed and maintained at the optimum level we describe. At the same time, as a biological strategy the use of beehives presents many challenges [44,47]. Specifically, (i) parasites diminish honey production and could also have a negative effect on the efficiency of beehives as elephant deterrents, (ii) bee stings could discourage people from practicing beekeeping; and (iii) the inability to maintain beehives at the optimum activity level could lead to a failure of the hive to deter elephants if people do not have the proper training. Given these challenges, people must master beekeeping to successfully use beehives, and more research is necessary to test the ability of hives to deter elephants in plantations rather than forests. Additional work is also necessary to render beehives an economically and biologically suitable conservation strategy.
